# Associations between Physical Activity and Health Parameters in Adolescent Pupils in Egypt

**DOI:** 10.3390/ijerph7041649

**Published:** 2010-04-12

**Authors:** Walid El Ansari, Said El Ashker, Laurence Moseley

**Affiliations:** 1 Faculty of Sport, Health & Social Care, University of Gloucestershire, Gloucester, GL2 9HW, UK; 2 Faculty of Sports and Physical Education, Mansoura University, Mansoura, Egypt; E-Mail: dr_said24@yahoo.com; 3 Faculty of Health, Sport, & Science, University of Glamorgan, Glyntaff Campus, Pontypridd, CF37 1DL, UK; E-Mail: lmoseley@glam.ac.uk

**Keywords:** physical activity, exercise, school pupils, children, obesity, health and fitness

## Abstract

Physical activity (PA) could be protective against hypertension, atherosclerosis, coronary heart disease and cardiovascular disease. This quantitative study assessed the association between a PA intervention and three anthropometric parameters (weight, body mass index, body fat) and four physiological parameters (cholesterol level, systolic blood pressure, diastolic blood pressure, heart rate) among secondary school pupils (N = 160) in Egypt through the school term. The pupils were randomised to an intervention group (80 pupils) and controls (80 pupils). Measurements were obtained for all pupils twice: at baseline; and then again after three months. The PA intervention programme comprised an ‘afterschool’ one hour of moderate exercise three times a week for three months. Both the controls and the intervention pupils attended the ‘normal’ exercise schedule provided by the school; in addition, the intervention group attended afterschool PA programme from about 2–3 o’clock in the afternoon. At baseline, employing pupil’s BMI, 27.5% and 28.8% of the intervention and control pupils respectively were classified as overweight. After three months, the percentage of overweight decreased to 12.5% in the intervention pupils, while it increased to 37.3% in the controls. At the end of the three months period, there were significant improvements across most anthropometric and physiological parameters of the intervention pupils when compared with the control children. The correlation coefficient of the improvements for the boys and the girls was 0.97, indicating clearly that the intervention was having nearly the same beneficial effect for boys and girls. A moderate PA programme for a modest period of 3 months could be effective in maintaining or enhancing pupil’s anthropometric and physiological parameters in comparison to the controls where there was deterioration in both parameters. Policy makers and secondary schools in Egypt might need to pay more attention to PA programmes conducted on school days, in order to motivate pupils to attend such programmes. There is also an urgent need to look at current PA systems within schools in Egypt in order to assess PA outside school times.

## 1. Introduction

The increase in body fat is a serious and widespread problem globally. There are about 350 million obese (body mass index, BMI ≥ 30 kg/m^2^) and 1 billion overweight persons (BMI ≥ 25), where ≈2.5 millions deaths are attributed to overweight/ obesity [1,2]. In the Eastern Mediterranean region, the highest levels of overweight persons (BMI ≥ 25) were in Kuwait, Egypt, United Arab Emirates, Saudi Arabia, Jordan and Bahrain, where the incidence of overweight/obesity for those aged ≥ 25 years was between 74%–86% (women) and 69%–77% (men) [3]. Unsurprisingly, in Egypt, the prevalence of hypertension (26.3% of adult population) is among the highest in the world [4].

Childhood obesity is a public health concern, particularly as obesity in childhood could lead to an increased likelihood of obesity later in life [5]. The global increase in obesity in children and adolescents could be attributed to decreased physical activity (PA), in addition to unhealthy lifestyle habits [6,7]. Five out of every eight children aged 9–13 in the United States do not participate in any PA during their non-school hours, and almost one fourth do not engage in any free-time PA [8]. Childhood obesity is associated with elevated blood pressure (BP), cholesterol, and BMI in childhood that could progress over time to adult premature cardiovascular disease (CVD) [9]. Indeed, there were significant differences in mean systolic and diastolic blood pressure between obese and non-obese Bahraini school children (aged 12–17 years), and children who have high blood pressure could be at greater risk of becoming hypertensive adults [10–12]. Because high blood pressure is important in the occurrence of coronary heart disease in adults [13], it is essential that the association between PA and blood pressure and other health parameters in adolescents be examined [14]. Current reports suggested that school-based PA interventions may be useful in the improvement of health parameters and lifestyle behaviours among children and adolescents which could lead to reduced CVD risk in adulthood [14].

The increased trend toward adiposity among adolescents in the Eastern Mediterranean region [15–18] places them at a high risk of adult obesity and its consequences in terms of chronic diseases later in life. Although the effects of adolescent obesity on blood pressure levels seem related to the accumulation of abdominal fat [19,20], few studies investigated the association of PA with health parameters in adolescent pupils in the Middle East [21]. For instance, little research has focussed on overweight/obesity among adolescent girls in the Eastern Mediterranean Region, even though studies elsewhere indicated an increasing prevalence and a growing health concern [16]. This is despite that in adults, the increasing prevalence of obesity and overweight were related to the risk of occurrence of coronary heart disease and hypertension [11,22].

Fortunately, obese/hypertensive adolescents can decrease their blood pressure through PA as an effective means for the prevention and treatment of obesity, hypertension and CVDs [23,24]. However, there are no recent studies that investigated the association of PA with anthropometric and physiological parameters in Egyptian adolescent school children. Such evidence base is required in order to design and implement PA intervention programmes in Egyptian schools.

### 1.1. Aim of the Study

This study assessed the relationships between a PA programme (the intervention) and health parameters in adolescent school pupils in Egypt. The three specific objectives were:

- Describe a range of anthropometric (weight, height, body mass index and body fat) and physiological (cholesterol level, systolic blood pressure, diastolic blood pressure and heart rate) parameters of a sample of Egyptian secondary school pupils;
- Assess the association between the PA intervention and pupils’ anthropometric parameters; and,
- Explore the association between the PA intervention and pupils’ physiological parameters.

## 2. Methods

### 2.1. Procedures and Participants

The study was approved by the Ethics Committee and Review Board of Mansoura University, Egypt. A little minority of schools in Mansoura city (situated in the Nile delta, Lower Egypt) have both indoor and outdoor sport facilities and sport equipment, hence an important criterion for the selection of the school where the study would be implemented was that it possessed sport facilities and sport equipment. One secondary school in Mansoura city was selected due to the availability of both indoor and outdoor sport facilities and sport kits at the school. This criterion allowed the PA programme (intervention) to be implemented irrespective of weather conditions, and also ensured the pupils’ safety. Pupils in the selected school were informed about the aims and objectives of the study, participation was voluntary, and those who agreed to participate could withdraw from the study if they wished. Participants were included in the study if they and their parents consented to participate.

The school’s physical education teacher invited all pupils (N = 450) to participate in the study, of which 200 pupils actually came forward to participate. These 200 pupils were roughly spread across the three years (Year 1, 2 and 3 pupils) of the secondary school. Each pupil was then provided with a consent form and a PA readiness questionnaire. The PA readiness questionnaire inquired about any medical problems or conditions that might exclude participants from the study; and also inquired about the participants’ favourite sport and PA activities. Pupils took home the consent form and the PA readiness questionnaire to complete with their parents. All pupils who participated in the study (and their parents) signed the forms. Based on the completed PA readiness questionnaires, 20 pupils were excluded because of reported medical condition/s. Hence, 180 pupils (70 boys, 110 girls) ‘passed’ the PA readiness questionnaire. As the number of girls exceeded the boys, of the 110 girls, 20 were randomly selected and assigned to be ‘reserve’ participants in case any of the confirmed participants wished to withdraw from the study. Of the remaining pupils (70 boys, 90 girls), the 70 boys were randomly assigned to either an intervention or control group; and the same was undertaken with the 90 girls. This procedure generated two groups each comprised 80 pupils (35 boys, 45 girls): the intervention group (80 pupils) participated in the PA programme (intervention); while the comparators (80 pupils) did not participate in the PA programme (controls). The mean age for the intervention group and controls were 15.7 years (SD 1.8 years) and 15.4 years (SD 1.6 years) respectively. All participants in the PA programme (intervention) agreed to attend and complete the PA programme and were medically fit to participate in the study *i.e.* in their PA readiness questionnaires, they reported not taking any medications for any chronic disease, and did not report any cardio-respiratory complaints.

For the 160 pupils, anthropometric (weight, height, BMI, body fat) and physiological (cholesterol, systolic blood pressure, diastolic blood pressure, heart rate) measurements were obtained twice: at baseline during the first school term (2007) (pre intervention); and after 3 months during the second school term (2007) (post intervention). At each of these two occasions, the second author together with research assistants (trained prior to the commencement of the study) obtained each of the measurements (detailed below) three times to ensure reliability. The research assistants were students from the Sport Training and Health Science Departments (University of Mansoura) who had experience in acquiring such measurements. Measurement techniques were revised by both the Heads of Sport Training and Health Science Departments at the University of Mansoura, in accordance with the American College of Sports Medicine Guidelines for exercise prescription and testing in order to ensure uniformity and accuracy [13].

### 2.2. PA Intervention

Generally this school provided two physical education lessons per week to the pupils, each of about 30 minutes duration. However, these physical education lessons could have benefitted from better supervision by more motivated teachers. Collectively, the lessons totalled 1 hour of PE per week, which is much less than international guidelines for PA for adolescents and also too little to yield health benefits. Furthermore, attendance at these physical education lessons was not compulsory *i.e.*, pupils could choose not to attend the lesson.

We employed a PA intervention premised on the United States Centres for Disease Control and Prevention (CDC) [8] guidelines to accumulate 60 minutes or more of moderate-intensity PA on most, and preferably all days of the week. The expected benefits to be accrued from such PA levels could contribute to preventing cardiovascular disease and decrease the death risk [25]. Pupils agreed that the time after school was a suitable time for the PA intervention; and that the 2–3 p.m. period was the most agreeable time to attend the PA programme. All pupils in the intervention group (35 boys, 45 girls) attended this afterschool PA intervention programme at the same time. A pre-requisite for the intervention pupils was that they regularly attend this sports and activities ‘package’ (*i.e.*, attendance was required at ≥90% of the sessions), and indeed all of the pupils attended more than the required attendance rate, which was recorded in an attendance sheet at each session.

Research assistants were trained prior to commencement of the study and delivered the PA programme to the intervention pupils. The PA intervention incorporated the favourable activities as indicated by the pupils in their responses to their favourite sport and PA activities in the PA readiness questionnaire. Hence the intervention included outdoor activities e.g. football (chosen by 18.7% of pupils), volleyball (12.5%), hand ball (13.7%), basketball (10%), cycling (5%), walking (6.25%), and small games (3.75%); and indoor activities (table tennis 8.75%), gym (7.5%), aerobic dance (10%), and aerobic boxing (3.75%). The PA intervention comprised three, 60-minute PA sessions each week for three months, delivering 180 minutes of PA per week, and the intensity of the physical exercise was controlled by maintaining the target heart rate in the moderate intensity zone (60–80% of the maximum HR). This is in agreement with the CDC guidelines [8] for children and adolescents, which comprises incrementally suitable and pleasurable variety of favourable activities.

### 2.3. Data Collection Tools

Each pupil was assigned a data capture sheet where research assistants recorded the pupil’s anthropometric and physiological parameters at baseline and after three months. Each of the measurements (detailed below) were obtained three times on the same day to ensure accuracy. The data sheet also included pupil’s age, school year and gender. All participants were informed not to intake any caffeine or tobacco on the day their physiological measurements were to be appraised; and not to perform any PA on the two days preceding the day when their physiological measurements were to be assessed. Anthropometric evaluation comprised three parameters:

*Weight (and height)*: measured using (Seca Digital Weight & Height Scale). Height was measured to the nearest 0.1 cm while the pupils stood barefooted, and body weight was measured to the nearest 0.1 kg while the pupils wore light clothing and no footwear.

*BMI Status*: calculated using Metric BMI Formula [BMI (kg/m^2^) = weight in kilograms/the squared height (m^2^)]. The BMI was employed to determine the number of pupils who were within normal weight (5th–85th percentile), overweight (85–95th percentile) and obese (>95th percentile) [26–30].

*Body fat*: skin folds thicknesses were measured using the Harpenden Skinfold Caliper [31,32]. In line with others [33–35] the 3-Site formula was used to measure skin folds (males: chest, abdomen, thigh; females: triceps, suprailiac, thigh). Body density was calculated according to standard formulae [Body density_♀_ = 1.0994921 − 0.0009929 (Sum of ‘triceps, suprailiac, thigh’ skin folds) + 0.0000023 (sum of ‘triceps, suprailiac, thigh’ skin folds)^2^ − 0.0001392(age); Body density_♂_ = 1.10938 − 0.0008267 (sum of ‘chest, abdomen, thigh’ skin folds) + 0.0000016 (sum of ‘chest, abdomen, thigh’ skin folds)^2^ − 0.0002574(age)]. Body fat percentage was described in line with the American College of Sports Medicine [13]. The values for three sites of the left side of the body were obtained in millimetres. Two research assistants worked as a team to measure all participants: one assistant pinched the skin at the measurement site to lift a double layer of skin and the underlying adipose tissue (not the muscle) and then applied the calipers 1 cm below and at right angles to the pinch; another research assistant took the reading within few seconds to write it in a record sheet. The two assistants took the measures at least three times and employed the average value in the particular formula.

Physiological measurements comprised four parameters: cholesterol was measured using Lifestream Cholesterol Monitor [36]. Such devices can be considered accurate for screening for elevated total cholesterol, and to accurately classify 80–90% of people into the appropriate risk category. Blood pressure and heart rate were measured using Omron Blood Pressure Monitor [37].

*Cholesterol*: we employed the Cholesterol classification in Children and Teenagers [acceptable normal (<170 mg); borderline (170–199 mg); or high (>200 mg)] [38]. Our participants were not fasting, in agreement with recommendations [39,40] that non fasting samples may be sufficient for cholesterol screening.

*Blood pressure*: using international guidelines [41,42], pupils were classified into ‘normal’, or those at risk of developing ‘high’ blood pressure, or those with ‘high’ blood pressure; [normal BP—both systolic blood pressure (SBP) and diastolic blood pressure (DBP) < 90th percentile; pre-hypertension —SBP and/or DBP ≥ 90th percentile but < 95th percentile or if BP exceeds 120/80 mmHg (even if <90th percentile for age, gender, and height); and hypertension defined as either SBP and/or DBP ≥ 95th percentile]. We measured BP on the same day on three separate occasions separated by 30 seconds; the average of three measurements was recorded [4].

*Heart rate*: heart rate (HR) was classified as described by others [43]: [‘good’ (<70_♂_ and <73_♀_ beats/minute respectively); ‘normal’ HR (71–77_♂_ and 74–80_♀_ beats/minute respectively); and ‘poor’ HR (>77_♂_ and >80_♀_ beats/minute respectively)].

Heart Rate and blood pressure readings were measured in a quiet room after ten minutes of rest. Both measurements were taken in the seated position with the right arm supported at heart level. A pediatric cuff was used. For the intervention pupils, two days of rest were provided after the last day of the 3 months PA programme before these measurements were undertaken.

### 2.4. Data Analysis

Data capture sheets obtained from the pupils were collated, cleaned and computer entered into SPSS v13.0. (SPSS Inc, Chicago). Spot checks on the spreadsheet were undertaken to ensure accuracy of data entry. The calculation of confidence limits and their statistical significance at the *p* < 0.05 level was undertaken using the CIA confidence intervals analysis program [44].

## 3. Results

### Were the two groups (intervention and control) similar at baseline?

**Figure 1.**
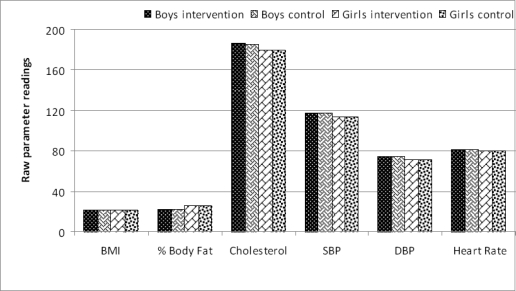
Mean values at baseline by gender: control and intervention pupils.

Yes, the two groups were similar at baseline. Table 1 shows the baseline anthropometric and physiological parameters for the intervention pupils and controls by gender. There were not any statistically significant differences between the intervention pupils and controls across all the anthropometric and physiological parameters. This is further confirmed by a visual examination of the values of the intervention pupils and controls across the seven parameters (Figure 1).

### Were the two groups (intervention and control) similar after 3 months?

**Figure 2.**
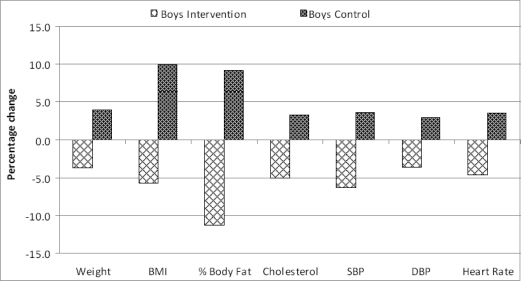
Changes in intervention and control pupils after 3 months: boys*. * All changes were significant at *p* < 0.05 (see Table 2).

**Figure 3.**
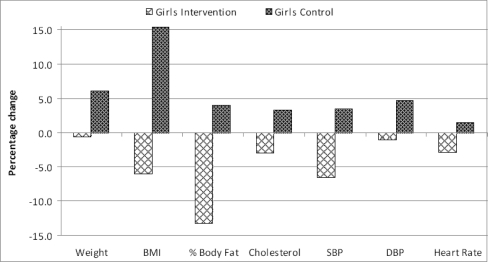
Changes in intervention and control pupils after 3 months: girls*. * All changes significant at *p* < 0.05, except % body fat (see Table 2).

No, the two groups were different after three months. Table 2 depicts the anthropometric and physiological parameters for the intervention and control pupils by gender three months later (one school term—the duration of the implemented PA intervention). Compared with the controls, the intervention pupils (both genders) achieved statistically significant decreases (improvements) in the levels of all the anthropometric and physiological parameters, except for the decreases in % body fat in girls. Table 2 and Figures 2 and 3 show the changes, after 3 months, for intervention and control pupils across the seven parameters for boys and girls respectively. The intervention group improved on all the assessed parameters, at a time when the control group were deteriorating on the same measures.

### Were the control pupils similar at baseline and after 3 months?

In most instances, the control pupils displayed increases (worsening) across all the anthropometric and physiological parameters that were examined. However these increases were only statistically significant for BMI in boys, and BMI and DBP in girls. Despite that lack of statistical significance in many parameters, it is noteworthy that for all the parameters and for both genders, the differences for the control pupils were positive (*i.e.*, the control pupils got worse). Table 3 shows the baseline measurements of the controls and their same measurements obtained three months later by gender.

### Were the intervention pupils similar at baseline and after 3 months?

With very few exceptions, the intervention pupils displayed decreases (improvements) in all their anthropometric and physiological parameters. However these decreases were only statistically significant for cholesterol and SBP in boys. The mean differences were in the desired direction on all the parameters measured (*i.e.*, improvements). Table 4 shows the pre- and post-intervention anthropometric and physiological parameters of the intervention pupils by gender.

### Intervention and control pupils’ health parameters after 3 months

*Weight*: intervention pupils’ mean weight decreased (from 61.2_♂_ kg to 58.9_♂_ kg, and from 60.2_♀_ kg to 59.8_♀_ kg) (Table 4). Both these weight decreases were not statistically significant. At the same time the controls’ mean weight increased (from 60.9_♂_ kg to 63.3_♂_ kg; from 61.1_♀_ kg to 64.8_♀_ kg, Table 3).

**Figure 4.**
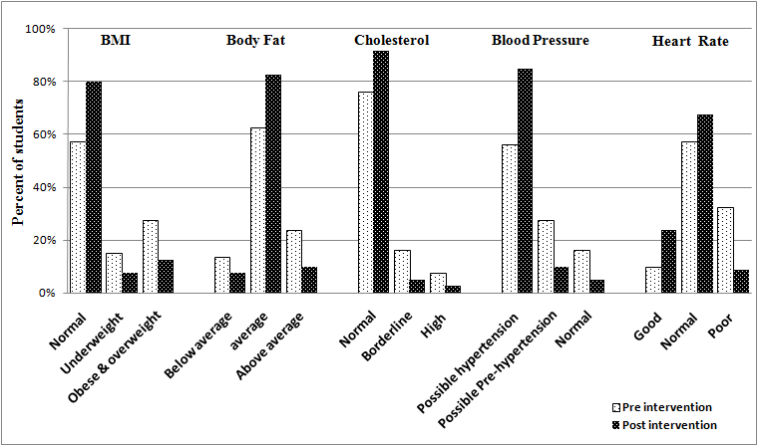
Anthropometric and physiological parameters of intervention pupils after 3 months.

**Figure 5.**
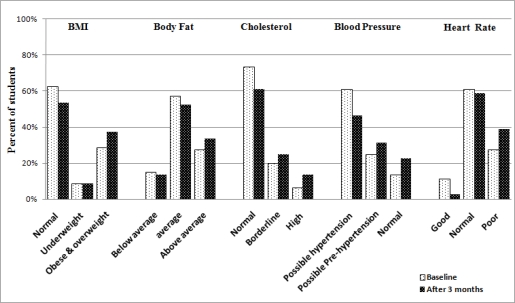
Anthropometric and physiological parameters of control pupils after 3 months.

*BMI*: the intervention pupils’ mean BMI decreased (from 20.9_♂_ to 19.7_♂_ kg/m^2^; from 21.6_♀_ to 20.3_♀_ kg/m^2^) (Table 4). Both these BMI declines were not statistically significant. During the same period, controls’ mean BMI significantly (*p* < 0.05) increased (from 21.2_♂_ to 23.3_♂_ kg/m^2^; from 21.4_♀_ to 24.7_♀_ kg/m^2^, Table 3). Further, within the intervention group 27.5% of pupils were classified as overweight or obese at baseline, which decreased to 12.5% after the PA intervention (Figure 4). Conversely, 28.8% of controls were categorized as overweight or obese at baseline, increasing to 37.3% after 3 months (Figure 5).

*Body fat*: intervention pupils’ mean % body fat decreased (from 22.1%_♂_ to 19.6%_♂_; from 25.6%_♀_ to 22.2%_♀_, Table 4). During the same period controls’ mean % body fat increased (from 21.9%_♂_ to 23.9%_♂_; from 25.1%_♀_ to 26.1%_♀_, Table 3). In addition, at baseline, 23.8% of intervention pupils were categorised as ‘above average' mean body fat percentage, which decreased to 10% after the PA intervention (Figure 4). Conversely, at baseline, 27.3% of controls were classified as ‘above average’ mean body fat percentage, increasing to 33.8% after 3 months (Figure 5).

*Cholesterol*: intervention pupils’ mean cholesterol level decreased significantly (*p* < 0.05) only in boys (from 186.5_♂_ to 177.2_♂_ mg/dl; from 179.4_♀_ to 174.1_♀_ mg/dl), while controls’ mean cholesterol increased (from 185.3_♂_ to 191.4_♂_ mg/dl; from 182.4_♀_ to 188.3_♀_ mg/dl, not significant) (Tables 3 and 4). Further, at baseline, 7.5% and 16.25% of intervention pupils were classified as having ‘high’ and ‘borderline’ cholesterol respectively, both decreasing to 2.5% and 5% respectively following the PA intervention. These decreases indicate that ≈17.8% of intervention pupils with initially high and borderline cholesterol had moved, after the intervention, to within the normal cholesterol range. Conversely, at baseline, 6.8% and 20.8% of controls exhibited ‘high’ and ‘borderline’ cholesterol respectively; after 3 months, these values increased to 13.8% and 25.8% respectively.

*Blood pressure*: Table 4 show that intervention pupils’ mean SBP decreased significantly (*p* < 0.05) only in boys (SBP from 117.2_♂_ to 109.7_♂_ mmHg). The other values showed decreases but were not statistically significant (DBP from 74.8_♂_ to 72.1_♂_ mmHg; SBP from 113.7_♀_ to 106.3♀ mmHg; DBP from 71.3_♀_ to 70.6_♀_ mmHg). Simultaneously, control pupils’ mean DBP increased significantly (*p* < 0.05) only in girls (DBP from 70.6_♀_ to 73.9_♀_ mmHg). The other values showed increases but were not statistically significant (SBP from 116.9_♂_ to 121.1_♂_ mmHg; DBP from 74.3_♂_ to 76.5_♂_ mmHg; SBP from 114.6_♀_ to 118.6_♀_ mmHg; (Table 3). At baseline, 16.3% and 27.5% of the intervention pupils were classified as being at risk of having increased blood pressure (*possible* ‘hypertension’ and ‘pre-hypertension’ respectively), which dropped to 5.3% and 9.8% subsequent to the PA intervention. These decreases translate to that ≈28.8% of the intervention pupils who were initially at risk of increased blood pressure were rendered, after the intervention, within the normal range. Conversely, at baseline, 13.8% and 25% of controls were at risk of having increased blood pressure (*possible* ‘hypertension’ and ‘pre-hypertension’ respectively), increasing to 22.5% and 31.25% after 3 months.

*Heart rate*: intervention pupils’ mean HR decreased (from 81.1_♂_ to 77.3_♂_ BPM; from 80.5_♀_ to 78.2_♀_ BPM, Table 4). In the same time controls’ mean HR increased (from 80.7_♂_ to 83.5_♂_ BPM; from 81.1_♀_ to 82.2_♀_ BPM) (Tables 3). Additionally, at baseline, 32.5% and 10% of intervention pupils were classified as ‘poor’ and ‘good’ HR respectively; after the PA intervention, ‘poor’ HR pupils dropped to 8.8 and ‘good’ HR pupils increased to 23.8% of the total number of intervention pupils (Figure 4). Conversely, 27.8% of controls had ‘poor’ HR and 11.25 % had ‘good’ HR at baseline; after 3 months ‘poor’ HR pupils increased to 38.8% and ‘good’ HR pupils decreased to 2.8% (Figure 5).

### Were the actual mean changes on each of the seven parameters similar for boys and girls?

**Figure 6.**
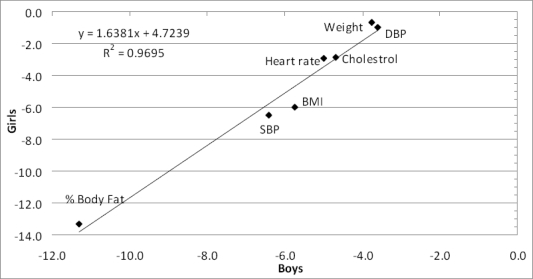
Correlation of percentage changes in mean parameter values: boys *versus* girls.

Figure 6 shows a plot of the relationship of the intervention group means by gender (boys *versus* girls) for the parameters under examination. It represents the extent of decrease on each parameter for the boys and for the girls. The correlation coefficient of the decreases for the boys and the girls was 0.97, indicating clearly that the intervention had the same effect, on the same parameters, for boys and girls. This provided more confidence that the relationship was probably real. For both genders, the parameters did not all decrease to the same extent: the % body fat was the parameter that decreased the most, while weight and DBP were the parameters that decreased the least. The extents of reductions of the remaining parameters were in between these two extremes.

A point to note is that whilst % body fat decreased relatively more, the actual weight decreased only a little (more or less stable body weight). This suggested that the decrease in % body fat was being simultaneously accompanied (replaced) by an increase in muscle mass, hence contributing to a stable weight.

## 4. Discussion

The PA intervention employed in the current study comprised 60 minutes, three times per week for three months. A criterion for participating in the PA intervention was that all pupils had to regularly attend the sessions and complete the period of the 3 months PA programme. Although the total amount of the intervention’s PA added up to 180 minutes per week (3 days × 60 minutes), which is slightly higher than the guideline recommendation of 150 minutes of moderate-intensity aerobic PA or 75 minutes of high-intensity aerobic PA per week to control weight [8], however we found mostly significant improvements in the parameters that were examined amongst the intervention pupils when compared to the controls who did not participate in the intervention. Starting with two very similar groups of pupils at baseline, three months later, the control group had deteriorated on all seven parameters while the intervention group had improved on all of them.

Risk factors associated with CVD (obesity, high percentage body fat, hypertension and cholesterol) set up themselves throughout childhood and could project into adulthood [45,46]. Many adolescents place their health at risk through lifestyles that comprise insufficient PA thus resulting in a high prevalence of obesity [47]. Certainly the overweight and obesity epidemic is spreading throughout the industrialised world with negative health effects, where for instance ≈55% of adult Americans reported a BMI > 25 kg/m^2^ [48]. These alarming trends are becoming evident in developing countries and could lead to high obesity levels in such countries if health risk behaviours continue. Indeed, the prevalence of obesity in Egypt is similar to that of developed nations (e.g., USA) [49]. The current study assessed the association of a PA programme on anthropometric and physiological parameters (weight, height, BMI, body fat, cholesterol, SBP, DBP and HR) in a sample of Egyptian adolescent pupils. Our findings suggested that school-based PA interventions may be useful in enhancing the health parameters among children and adolescents. This could be in turn interpreted as reduced risk of CVD in adulthood [14].

In relation to the first objective of the study, we described a range of anthropometric and physiological parameters for a sample of secondary school pupils. School-based BMI measurement is important as it is a potential approach to address obesity among youth [50]. Objective measurement of weight and height are preferred than reported values, as overall self-reported measurements might systematically underestimate weight and BMI [51,52]. Since the classification into BMI-categories using self-reported adolescents’ height and weight are not very accurate, we based our BMI calculation on objective measurements [53–56].

Out of 160 pupils initially measured, 25% of the samples were overweight or obese at baseline (asserted by their BMI). This baseline level compared unfavourably with levels from elsewhere; ≈15% of adolescent girls in Norway, and 19% in Argentina (15–18 year old) were overweight or obese [57]. This baseline level was also higher than the 13.4% (14–18 year olds) who were reported overweight or obese in a previous study in Egypt [58]. Indeed, Egyptian girls’ mean BMI was higher than children of comparable ages in other Arab countries of [16], and our sample’s mean BMI (21.28, data not presented) was slightly higher than mean BMI (19.2) of 404 pupils in Japan [59]. Current adolescent overweight will likely lead to large future economic and health burdens, especially lost productivity from premature death and disability [60]. Likewise, the baseline mean % body fat for our sample (22.0_♂_, 25.4_♀_, data not presented) was a little higher than that of 327 Brazilian adolescent pupils (18.1_♂_, 23.8_♀_ %) [61].

With reference to baseline mean SBP, our sample’s values (116.9_♂_–114.6_♀_ mmHg_controls_; 117.2_♂_–113.7_♀_ mmHg_intervention pupils_, Table 1) were slightly higher than the 107.1 mmHg reported in 2,156 Argentinean adolescents (15 to < 18 years old), but close to those of 676 adolescents in Norway (119.9 mmHg) [57]. Similarly, our baseline mean DBP (74.3_♂_–70.6_♀_ mmHg_controls_; 74.8_♂_–71.3_♀_ mmHg_intervention pupils_, Table 1) were slightly higher than Norway and Argentina (64 and 67 mmHg respectively). At baseline ≈16% of our sample was at risk of increased blood pressure, in comparison to Bahraini adolescents where 14% were classified as having high blood pressure [10]. Hypertension is becoming common among Egyptians, confirmed by Galal’s [58] findings that the prevalence of hypertension among Egyptians is very high, particularly among women. This is unsurprising given that the increasing rates of hypertension in children and adolescents could be correlated to the risk of coronary artery disease in adulthood [62].

As regards to baseline mean cholesterol levels (Table 1), our sample’s lowest (179.4_♀_ mg/dl_intervention_) and highest (186.5_♂_ mg/dl_intervention_) values were both higher than those of 13 years old pupils in Japan (170.1 mg/dl) [59]; and also much higher than levels (137.4–141.7 mg/dl) of 327 Brazilian adolescents [61]. Finally, in connection with the baseline mean HR, our sample’s lowest (80.5_♂_ BPM_intervention_) and highest (81.2_♀_ BPM_controls_) values were close to those of Brazilian children aged 9–11 years (84.8 BPM in obese and 80.2 BPM in non-obese children) [63].

In relation to the study’s second objective pertaining to anthropometric parameters, the comparisons of our intervention group with controls suggested a positive relationship between a moderate PA intervention and averting obesity and becoming unfit (e.g., decreased BMI and % body fat values, Table 2). This supported proposals that PA could defend against weight gain by increased energy expenditure [64]. Theoretically, although there is the potential for obese adolescents to lose weight and maintain their weight loss by participating in regular PA (at least one hour three times per week), it is rarely achievable to keep the weight off without participating in PA programmes [65]. The positive and significant association between the PA intervention and decreased BMI in our sample of boys and girls is shown in Table 2. This is in agreement with Gamble *et al*. [66]: a factor that has been consistently related to childhood BMI status is PA.

The results of the present study add to the increasing evidence that demonstrate the benefits of regular PA as a management plan for tackling obesity in adolescents. Our findings agree with others where regular PA was associated with significant reductions (improvement) in obesity [67]. After our PA intervention, % body fat in the intervention boys was significantly lower when compared with controls after 3 months (Table 2). Studies have reported significant improvements in % body fat following programmes that included PA [14]. Therefore, the combination of increasing levels of PA and avoidance of gain in fat mass is likely to be a successful approach for preventing cardiovascular and metabolic disease [68].

In relation to the study’s third objective (physiological parameters), we calculated BP as the mean of three consecutive measurements. This is important, as multiple BP measurements are required to suggest the risk of increased blood pressure [69]. We found a positive association between PA intervention and the SBP and DBP values (Table 2). Hypertension is a major public health problem worldwide [70]. There is a positive association between BP and % body fat in adolescents [65], supported by that during 3 months of PA, when compared to controls, our intervention pupils’ SBP and DBP decreased (SBP differences of 11.4_♂_ and 12.3_♀_ mmHg; DBP differences of 4.4_♂_ and 3.3_♀_ mmHg_♀_) respectively, along with decrease in body fat (differences of 4.3%_♂_ and 3.9%_♀_). However the decrease in % body fat in girls did not reach statistical significance (Table 2).

In connection with cholesterol, by comparing the post intervention values of the intervention pupils with control pupils after 3 months (Table 2), our findings suggested that for boys and for girls, the PA intervention was associated with decreases in cholesterol levels. These findings are in agreement with the U.S. Department of Health and Human Services [71] that suggest that one approach for maintaining optimal cholesterol levels in children and adolescents is exercise; a low-cost, non-pharmacologic intervention available to the vast majority of children and adolescents.

As regards resting heart rate, the effects of physical exercise on HR are not unequivocal. For instance, in middle aged men, intermediate intensity exercise is associated with positive modification in HR [72]. Conversely, few studies reported influence of physical training on HR [73] in middle-aged people, and others similarly reported that high intensity interval training during 7 weeks did not affect heart rate in 10-year old children [73]. In contrast with others (e.g., Gamelin *et al.* [74]), we found an association between PA and HR for both genders. This contrast might be explained by the fact that our PA intervention was of longer duration (three, one-hour regular training sessions each week for 3 months) thus delivering about 70% more PA ‘volume’ than Gamelin *et al.* [74] (7 weeks of three 30-min sessions), hence providing more time in order to impact on HR.

As regards the control children, there were significant increases (worsening) when compared with intervention pupils across many of their parameters after 3 months. It is possible to conceive an association between a low PA level (the ‘low level’ physical education delivery that is normally provided by the school) and the worsening anthropometric and physiological parameters in the controls. Unhealthy habits could be formed at this age regarding nutritional lifestyle and low PA levels and could contribute negatively to their anthropometrical and the physiological parameters. This suggested that in the social contexts of pupils, schools that do not adequately provide PA sufficient for health benefits might instead become effective environments for the propagation of negative lifestyles.

Regular attendance of moderate-intensity level PA could result in better anthropometric and physiological parameters in children. Various PA interventions have been implemented with different populations to examined their associations with different anthropometric, physiological, and body composition parameters [75,76]. Some of this research supported our findings that moderate-intensity PA might be useful to decrease the hazard of heart disease in low active individuals [77].

Perhaps a primary strategy for improving the long-term health of children and adolescents through exercise may be creating lifestyle patterns of regular PA that carry over to the adult years. Our findings are consistent with others [78], overweight and hypertensive adolescents can reduce their blood pressure through PA, particularly if they lose weight. Such benefits of regular PA, if popularised and scaled up to be implemented as part of integrated community-wide intervention programmes, could play a major role in the eastern Mediterranean Region in helping to raise community awareness and involve people in health promotion and disease prevention [79]. However, we assume that the lack of sport equipment at schools, as well as lack of exercise facilities and appropriate sport playgrounds could be some of the barriers to regular PA the Egyptian children. Our study suggested that an appropriately tailored PA intervention programme of sufficient duration could offer school pupils activities that interest them, and hence they would be motivated to participate. However more efforts are required in Egypt for the promotion of PA so that children and adolescents can avoid the risk factors associated with a range of non-communicable chronic diseases.

This study has limitations. It is cross sectional hence findings are associations, not causations. One school in north Egypt (Delta region) was selected for the study. The results would therefore need to be affirmed by bigger studies incorporating more schools in the other regions in Egypt in order to be adequately powered statistically. Not many schools in Egypt have both indoor and outdoor sport facilities and appropriate sport equipment facilities, hence factors such as weather and equipment may be major impediments to the pilot testing and/or expansion of this programme. Due to these factors, the generalisability of this PA programme to other schools in Egypt would need to exercise caution. The sample comprised 160 adolescents out of 450 children in the school who volunteered to participate, representing about 40% volunteer rate. We are unable to assess how those who volunteered could have been different on the measured parameters from those who did not volunteer. Due to funding constraints, other possible factors that could have contributed to our findings were not measured (e.g., diet control and/or extra ‘workouts’ that the intervention pupils who participated in the intervention PA might have undertaken). The PA intervention we employed delivered 180 minutes of PA per week, which is slightly less than the maximum 210 minutes per week recommended by some authorities [The United States Centres for Disease Control and Prevention 2001]. We undertook the blood pressure measurements on one day and not on three consecutive days, which is required for an accurate diagnosis of hypertension. Future research needs to confirm the prevalence of obesity, overweight and other health parameters in other larger and different samples in Egypt (younger children and older adolescents); to understand the barriers to and motivators/ promoters of exercise in and outside schools; and, to measure other possible confounding factors that could impact on the parameters that were measured.

## Figures and Tables

**Table 1.** Baseline anthropometric and physiological parameters of control and intervention pupils by gender.

**Parameter**	**Control Mean (SD)**	**Intervention Mean (SD)**	**Difference*a***	**95% Confidence Limits**		**P - value**
				**Min**	**Max**	
**Boys**						

**BMI†**	21.2 (3.6)	20.9 (4.1)	+0.3	−1.45	+2.05	NS
**% Body Fat**	21.9 (5.4)	22.1(6.3)	−0.2	−2.86	+2.46	NS
**Cholesterol§**	185.3 (18.3)	186.5 (17.1)	−1.2	−9.11	+6.71	NS
**SBP‡**	116.9 (10.2)	117.2 (8.9)	−0.3	−4.56	+3.96	NS
**DBP#**	74.3 (7.2)	74.8 (6.8)	−0.5	−3.63	+2.63	NS
**Heart RateΨ**	80.7 (7.6)	81.1(8.2)	−0.4	−3.96	+3.16	NS

**Girls**						

**BMI†**	21.4 (3.8)	21.6 (4.5)	−0.2	−2.09	+1.69	NS
**% Body Fat**	25.1 (8.1)	25.6 (7.4)	−0.5	−3.99	+2.99	NS
**Cholesterol§**	182.4 (14.3)	179.4 (19.5)	+3.0	−4.82	+10.82	NS
**SBP‡**	114.6 (11.3)	113.7 (9.1)	+0.9	−3.64	+5.44	NS
**DBP#**	70.6 (6.1)	71.3 (5.9)	−0.7	−3.39	+1.99	NS
**Heart RateΨ**	81.2 (9.1)	80.5 (7.3)	+0.6	−3.05	+4.25	NS

† BMI =body mass index (kg/m^2^),

§ in mg/dl;

‡ SBP = systolic blood pressure (mmHg);

# DBP = diastolic blood pressure (mmHg);

Ψ beats per minute (BPM); NS = not significant;

a = control mean minus intervention mean.

**Table 2.** Anthropometric and physiological parameters of control and intervention pupils by gender after 3 months.

**Parameter**	**Control Mean (SD)**	**Intervention Mean (SD)**	**Difference*a***	**95% Confidence Limits**		**P-value**
				**Min**	**Max**	
**Boys**						

**BMI†**	23.3 (3.5)	19.7 (3.1)	+3.6	+2.12	+5.07	<0.05
**% Body Fat**	23.9 (10.1)	19.6 (8.2)	+4.3	+0.23	+8.37	<0.05
**Cholesterol§**	191.4 (19)	177.2 (19.3)	+14.2	+5.60	+22.80	<0.05
**SBP‡**	121.1 (8.3)	109.7 (7.6)	+11.4	+7.85	+14.95	<0.05
**DBP#**	76.5 (7.2)	72.1 (6.8)	+4.4	+1.27	+7.53	<0.05
**Heart RateΨ**	83.5 (8.3)	77.3 (9.5)	+6.2	+2.16	+10.24	<0.05

**Girls**						

**BMI†**	24.7 (3.7)	20.3 (3.6)	+4.4	+2.77	+6.04	<0.05
**% Body Fat**	26.1 (7.9)	22.2 (9.8)	+3.9	–0.15	+7.95	NS
**Cholesterol§**	188.3 (16.5)	174.1 (20.2)	+14.2	+5.82	+22.58	<0.05
**SBP‡**	118.6 (7.5)	106.3 (8.3)	+12.3	+8.73	+15.87	<0.05
**DBP#**	73.9 (6.1)	70.6 (5.9)	+3.3	+0.61	+5.99	<0.05
**Heart RateΨ**	82.2 (9.8)	78.2 (7.6)	+4	+0.13	+7.87	<0.05

† BMI = body mass index (kg/m^2^),

§ in mg/dl;

‡ SBP = systolic blood pressure (mmHg);

# DBP = diastolic blood pressure (mmHg);

Ψ beats per minute (BPM), NS = not significant;

a = control mean minus intervention mean.

**Table 3.** Anthropometric and physiological parameters at baseline and 3 months later of control pupils by gender.

**Parameter**	**Baseline Mean (SD)**	**After 3 months Mean (SD)**	**Difference*a***	**95% Confidence Limits**		**P-value**
				**Min**	**Max**	
**Boys**						

**Weight^¥^**	60.9 (9.3)	63.3 (8.9)	+2.4	–1.9	+6.7	NS
**BMI†**	21.2 (3.6)	23.3 (3.5)	+2.1	+0.4	+3.8	<0.05
**% Body Fat**	21.9 (5.4)	23.9 (10.1)	+2.0	–1.9	+5.9	NS
**Cholesterol§**	185.3 (18.3)	191.4 (19)	+6.1	–2.8	+15.0	NS
**SBP‡**	116.9 (10.2)	121.1 (8.3)	+4.2	–0.2	+8.6	NS
**DBP#**	74.3 (7.2)	76.5 (7.2)	+2.2	–1.2	+5.6	NS
**Heart RateΨ**	80.7 (7.6)	83.5 (8.3)	+2.8	–1.0	+6.6	NS

**Girls**						

**Weight^¥^**	61.1 (11)	64.8 (11.4)	+3.7	–1.6	+9.0	NS
**BMI†**	21.4 (3.8)	24.7 (3.7)	+3.3	+1.5	+5.1	< 0.05
**% Body Fat**	25.1 (8.1)	26.1 (7.9)	+1.0	–2.8	+6.5	NS
**Cholesterol§**	182.4 (14.3)	188.3 (16.5)	+5.9	–1.5	+12.3	NS
**SBP‡**	114.6 (11.3)	118.6 (7.5)	+4.0	–0.6	+8.7	NS
**DBP#**	70.6 (6.1)	73.9 (6.1)	+3.3	+0.4	+6.2	< 0.05
**Heart RateΨ**	81.1 (9.1)	82.2 (9.8)	+1.1	–3.4	+5.6	NS

¥ In Kilograms;

† BMI = body mass index (kg/m^2^),

§ in mg/dl;

‡ SBP = systolic blood pressure (mmHg);

# DBP = diastolic blood pressure (mmHg);

Ψ beats per minute (BPM);

a = mean after 3 months minus baseline mean.

**Table 4.** Pre and post-intervention anthropometric and physiological parameters of intervention pupils by gender.

**Parameter**	**Pre Mean (SD)**	**Post Mean (SD)**	**Difference*a***	**95% Confidence Limits**		**P - value**
				**Min**	**Max**	
**Boys**						

**Weight^¥^**	61.2 (8.3)	58.9 (9.2)	−2.3	−6.9	+1.4	NS
**BMI†**	20.9 (4.1)	19.7 (3.1)	−1.2	−2.7	+0.3	NS
**% Body Fat**	22.1 (6.3)	19.6 (8.2)	−1.5	−5.6	+0.5	NS
**Cholesterol§**	186.5 (17.1)	177.2 (19.3)	−9.3	−16.9	–1.7	<0.05
**SBP‡**	117.2 (8.9)	109.7 (7.6)	−7.5	−11.0	–4.0	<0.05
**DBP#**	74.8 (6.8)	72.1 (6.8)	−2.7	−5.5	+0.1	NS
**Heart RateΨ**	81.1 (8.2)	77.3 (9.5)	−3.8	−7.6	+0.1	NS

**Girls**						

**Weight^¥^**	60.2 (9.5)	59.8 (10.2)	−0.4	−4.5	+3.7	NS
**BMI†**	21.6 (4.5)	20.3 (3.6)	−1.3	−3.0	+0.4	NS
**% Body Fat**	25.6 (7.4)	22.2 (9.8)	−3.4	−7.0	+0.2	NS
**Cholesterol§**	179.4 (19.5)	174.1 (20.2)	−5.3	−13.6	+3.0	NS
**SBP‡**	113.7 (9.1)	106.3 (8.3)	−7.4	−11.0	+3.8	NS
**DBP#**	71.3 (5.9)	70.6 (5.9)	−0.7	−3.2	+1.8	NS
**Heart RateΨ**	80.5 (7.3)	78.2 (7.6)	−2.3	−5.4	+0.8	NS

¥ In Kilograms;

† BMI = body mass index (kg/m^2^),

§ in mg/dl;

‡ SBP = systolic blood pressure (mmHg);

# DBP = diastolic blood pressure (mmHg);

**Ψ** beats per minute (BPM);

a = post-intervention mean minus pre-intervention mean.
